# A Plasma Biomarker Signature of Immune Activation in HIV Patients on Antiretroviral Therapy

**DOI:** 10.1371/journal.pone.0030881

**Published:** 2012-02-17

**Authors:** Anupa Kamat, Vikas Misra, Edana Cassol, Petronela Ancuta, Zhenyu Yan, Cheng Li, Susan Morgello, Dana Gabuzda

**Affiliations:** 1 Department of Cancer Immunology and AIDS, Dana Farber Cancer Institute, Boston, Massachusetts, United States of America; 2 Harvard Medical School, Boston, Massachusetts, United States of America; 3 Department of Microbiology and Immunology, Faculty of Medicine, CHUM-Research Center, Université de Montréal, Montreal, Canada; 4 Department of Biostatistics, Dana Farber Cancer Institute, Boston, Massachusetts, United States of America; 5 Mount Sinai Medical Center, New York, United States of America; Ghent University, Belgium

## Abstract

**Background:**

Immune activation is a strong predictor of disease progression in HIV infection. Combinatorial plasma biomarker signatures that represent surrogate markers of immune activation in both viremic and aviremic HIV patients on combination antiretroviral therapy (cART) have not been defined. Here, we identify a plasma inflammatory biomarker signature that distinguishes between both viremic and aviremic HIV patients on cART and healthy controls and examine relationships of this signature to markers of disease progression.

**Methods:**

Multiplex profiling and ELISA were used to detect 15 cytokines/chemokines, soluble IL-2R (sIL-2R), and soluble CD14 (sCD14) in plasma from 57 HIV patients with CD4 nadir <300 cells/µl and 29 healthy controls. Supervised and unsupervised analyses were used to identify biomarkers explaining variance between groups defined by HIV status or drug abuse. Relationships between biomarkers and disease markers were examined by Spearman correlation.

**Results:**

The majority (91%) of HIV subjects were on cART, with 38% having undetectable viral loads (VL). Hierarchical clustering identified a biomarker cluster in plasma consisting of two interferon-stimulated gene products (CXCL9 and CXCL10), T cell activation marker (sIL-2R), and monocyte activation marker (sCD14) that distinguished both viremic and aviremic HIV patients on cART from controls (p<0.0001) and were top-ranked in variables important in projection plots. IL-12 and CCL4 were also elevated in viremic and aviremic patients compared to controls (p<0.05). IL-12 correlated with IFNα, IFNγ, CXCL9, and sIL-2R (p<0.05). CXCL10 correlated positively with plasma VL and percentage of CD16+ monocytes, and inversely with CD4 count (p = 0.001, <0.0001, and 0.04, respectively).

**Conclusion:**

A plasma inflammatory biomarker signature consisting of CXCL9, CXCL10, sIL-2R, and sCD14 may be useful as a surrogate marker to monitor immune activation in both viremic and aviremic HIV patients on cART during disease progression and therapeutic responses.

## Introduction

Chronic immune activation is a hallmark of HIV disease that is strongly linked to disease progression [1], [2]. Markers of immune activation in both treated and untreated HIV-infected patients include enhanced expression of activation markers on peripheral blood T cells, B cells, monocytes, dendritic cells, and natural killer (NK) cells, and increased levels of inflammatory cytokines and chemokines [1], [3], [4], [5], [6], [7]. The causes of immune activation in HIV infection are poorly understood, but are likely to be multifactorial and include persistent elevation of type I and II interferons (IFN), innate and adaptive immune responses to HIV infection and bacterial products that translocate from a leaky gut, direct effects of HIV virions and/or viral proteins, co-infections with non-HIV pathogens, non-antigen specific bystander activation of immune cells, and dysregulated cytokine and chemokine production [1], [3], [4], [8], [9]. The innate immune system responds immediately during the course of HIV infection through production of type I and II IFN and other cytokines, and viral replication correlates with upregulation of type I IFN-stimulated genes. Type 1 interferons, produced primarily by plasmacytoid dendritic cells (pDC), along with activated monocytes and T cells, play a central role in mediating persistent inflammation in HIV infection [3], [10], [11], [12]. Despite extensive study, the mechanisms driving persistent induction and dysregulation of interferon responses and chronic inflammation are poorly understood.

Despite years of suppressive combined antiretroviral therapy (cART) in patients with undetectable plasma viral loads (VL) and CD4 reconstitution to normal or near normal levels, low levels of immune activation persist in treated HIV infection. Elevated circulating levels of activated immune cells [13], [14], [15], [16], [17], soluble activation markers (e.g., sTNFR, sCD27, sCD40L, sCD14, type I/II IFN, CCL2, CCL4, C-reactive protein, and D-dimer) [7], [18], [19], [20], [21], [22], [23], [24], [25], and markers of microbial translocation [9], [26] have been used to study relationships between immune activation and clinical outcomes in patients on cART. A number of studies have focused on identifying biomarkers to monitor HIV immunopathogenesis in patients on cART, yet no single marker or combinatorial biomarker signature has proved to be reliable for diagnostic or therapeutic purposes. An inflammatory biomarker signature that can distinguish a broad spectrum of HIV patients with suppressed or nonsuppressed VL on cART from uninfected healthy controls may be useful as a surrogate marker to monitor chronic immune activation during disease progression and therapeutic responses.

In this study, we performed multiplex cytokine/chemokine profiling and used supervised and unsupervised classification methods to identify a plasma inflammatory biomarker signature that represents a potential surrogate marker of immune activation in both viremic and aviremic HIV patients on cART. Because the cohort had a high frequency of drug abuse, we also evaluated the effects of active cocaine use on biomarker expression. We identified a plasma biomarker signature consisting of 2 interferon-stimulated gene products (CXCL9 and CXCL10), T cell activation marker (sIL-2R), and monocyte activation marker (sCD14) that distinguishes viremic and aviremic HIV patients on cART from uninfected healthy controls. This biomarker signature may serve as a surrogate marker of immune activation that may prove useful for monitoring disease progression and therapeutic responses in HIV patients on cART with diverse clinical phenotypes.

## Methods

### Ethics statement

All HIV+ subjects were enrolled with written informed consent and IRB approval at each study site (Lemuel Shattuck Hospital, Manhattan HIV Brain Bank, National Neurological AIDS Bank, California NeuroAIDS Tissue Network, Texas NeuroAIDS Research Center). The IRB at Dana-Farber Cancer Institute approved the research involving HIV+ subjects as exempt because samples and clinical data were provided without any patient or donor identifiers. HIV/HCV-negative control plasma samples from healthy control subjects were purchased from Bioreclamation LLC or Research Blood Components (Brighton, MA), or were collected from HIV-negative healthy volunteers at Dana-Farber Cancer Institute with written informed consent and IRB approval from Dana-Farber Cancer Institute.

### Subjects

HIV+ subjects (n = 57) with CD4 nadir <300 cells/µl and relatively low CD4 counts (median 81, range 3–688 cells/µl) were recruited at the Lemuel Shattuck Hospital (n = 26), and at four sites (Manhattan HIV Brain Bank, National Neurological AIDS Bank, California NeuroAIDS Tissue Network, Texas NeuroAIDS Research Center) in the National NeuroAIDS Tissue Consortium (NNTC; n = 26) or CNS HIV Anti-Retroviral Therapy Effects Research (CHARTER; n = 5). The majority (91%) were on cART. Plasma HIV RNA levels were log_10_ transformed for statistical analysis. Undetectable plasma VL values were assigned a log_10_ value of 2.6 (400 HIV RNA copies/ml), reflecting the sensitivity cutoff of the assay most widely used during these assessments; values below this cutoff reflect lower assay sensitivity (25 or 50 copies) for some sites. Current substance abuse (within 30 days of plasma sampling) was determined by the Psychiatric Research Interview for Substance and Mental Disorders (PRISM) [27] or Composite International Diagnostic Interview (CIDI) [28] and urine toxicology at time of blood draw. HIV/HCV-negative control plasma samples from healthy control subjects (n = 29) were purchased from Bioreclamation LLC (n = 13) or Research Blood Components (Brighton, MA; n = 2), or were collected from normal HIV-negative healthy volunteers at Dana-Farber Cancer Institute (n = 14).

### Cytokine/chemokine profiling by multiplex assay

A multiplex immunoassay (Bio-source 25-plex Human Cytokine Assay; Invitrogen) was used according to the manufacturer's instructions to measure levels of IL-1RA, IL-1*β*, IL-2, IL-4, IL-5, IL-6, IL-7, IL-8, IL-10, IL-12, IL-13, IL-15, IL-17, IFN-α, IFNγ, TNF, sIL-2R, GM-CSF, CCL2/MCP-1, CCL3/MIP-1α, CCL4/MIP-1*β*, CXCL10/IP-10, CXCL9/MIG, CCL11/eotaxin, and CCL5/RANTES. Briefly, plasma samples were diluted 1∶2 with assay buffer, and incubated with antibody-coupled beads. Complexes were washed, incubated with biotinylated detection antibody, and subsequently with streptavidin-phycoerythrin, prior to assessing titers of cytokine concentration. Standard curves run in duplicate were established with recombinant cytokines. Analyte levels were determined using a multiplex array reader from Luminex™ Instrumentation System (Bio-Plex Workstation from Bio-Rad); plasma samples were run in duplicate and analyte concentrations were calculated as the average of two independent measures using Bioplex Manager Software.

### Quantification of soluble CD14 using ELISA

Levels of soluble CD14 in plasma samples were measured using a commercial assay (Quantikine ELISA kit, R & D systems).

### Flow cytometry analysis of monocyte subsets

PBMC were isolated from fresh peripheral blood samples from 20 HIV+ subjects from the Lemuel Shattuck Hospital by Ficoll-Paque centrifugation and monocyte subsets analyzed by FACS analysis as described [29]. Fluorochrome-conjugated Abs used for FACS analysis were CD14, CD16, CD19, CD33, CD16b, CD66b, CD56, and CD3 (Beckman Coulter); CD4, CCR5, CD69, and HLA-DR (BD Pharmingen). To quantify CD16+ monocytes, PBMC depleted of CD3^+^ T-cells were stained with PE-CD14 and PE-Cy5 CD16 Abs and FITC-conjugated Abs against T-cell (CD3), granulocyte (CD16b/CD66b), B cell (CD19), NK cell (CD56), and dendritic cell (CD1c) markers. CD16+ monocytes (CD14+/CD16+) were distinguished from granulocytes by HLA-DR and lack of CD16b/CD66b expression, and from NK cells by higher forward and side scatter characteristics, CD14, CD4, and CD33, and lack of CD56 expression

### Data processing, normalization, and analysis

Raw values for each analyte were normalized to the mean of healthy controls, log_2_-transformed, and then subjected to supervised or unsupervised hierarchical clustering. The raw data, normalized values, and clinical data for each sample are included as supplemental data (Table S1). Cytokines with >20% missing data were excluded from further analysis; hence, analysis was performed for 16 biomarkers represented in the 25-plex assay in addition to sCD14 measured by ELISA. The lower limit of detection (LOD) for each biomarker was specified by the manufacturer for each lot number. For imputation of missing (non-detected) values, the LOD was used to replace missing values. For one analyte (IL-15), missing values were imputed in lowest-value mode as described [30] using the lowest detected value (LDV), corresponding to the most frequently occurring value (mode) among detectable values below the LOD. In this instance, missing values were replaced using the LDV rather than the LOD because the LDV was observed for a significant percentage of samples (15%) and the coefficient of variance for these observations (9.7%) indicated the measurement was reproducible. We chose this approach for imputation because it is conservative, reducing the probability of artificially creating differential expression or correlations. Hierarchical clustering was performed with dChip software using Euclidean distance and average linkage to analyze plasma biomarker levels across HIV subjects and healthy controls. Comparison criteria required the fold change (FC) between group means to exceed a specific threshold (FC≥1.3) and mean difference to be statistically significant by unpaired t-test (p<0.05 with false discovery rate (FDR) controlled at <5%). Principal component analysis (PCA) and partial-least squares discriminant analysis (PLS-DA) were performed on the Metaboanalyst web portal [31] using normalized and autoscaled expression values. The web portal utilizes the prcomp function of the stats package, and plsr function of the pls package of R, respectively. Class labels were permuted 2000 times to test whether differences between groups are significant. For every PLS-DA model built, the sum of squares between/sum of squares within (i.e., the B/W ratio) was computed and displayed in a histogram of random class assignments [32]. To examine relationships between normalized cytokine expression values and clinical covariates, Spearman correlations were run using the correlation test function in the stats package of R version 2.10.1 (http://www.r-project.org/).

## Results

### Plasma biomarker cluster consisting of CXCL9, CXCL10, sIL-2R, and sCD14 distinguishes HIV subjects from controls

The study cohort consisted of 57 HIV subjects with advanced disease (CD4 nadir <300; median, 61 cells/µl; range, 1–261 cells/µl), relatively low CD4 counts (median, 81 cells/µl; range, 3 – 688 cells/µl), variable plasma HIV RNA levels (median 2,460 copies/ml; range, undetectable – 2,210,000 copies/ml), and high frequency of recent substance abuse (70%) and HCV co-infection (47%) (Table 1). The majority were on cART (91%), with 38% having undetectable plasma VL (<400 HIV RNA copies/ml). HIV/HCV-negative subjects were used as healthy controls (n = 29).

**Table 1:** Demographic and clinical characteristics of HIV+ subjects in the study cohort.

**Age (years)**	
Mean ± SD	45±7.3
Median (range)	44 (32–63)
**Gender**	
Male	43 (75%)
Female	14 (25%)
**Race**	
African American	25 (44%)
Caucasian	15 (26%)
Hispanic	14 (25%)
Other	3 (5%)
**Risk factor**	
Sexual transmission	17 (30%)
Intravenous drug abuse	40 (70%)
**CD4 T cell count (cells/µl)**	
Mean ± SD	122±126
Median (range)	81 (3–688)
**CD4 nadir T cell count (cells/µl)**	
Mean ± SD	82±69.1
Median (range)	61 (1–261)
**Plasma HIV RNA (copies/ml)**	
Mean ± SD	131,365±408,505
Median (range)	2460 (<50–2,210,000)
<400 copies/ml	22 (38%)
**cART**	
Yes	52 (91%)
No	5 (9%)
**HCV co-infection**	
Positive	27 (47%)
Negative	18 (32%)
Unknown	12 (21%)
**Substance abuse**	
Opiates*	22 (38%)
Cocaine*	32 (55%)
None	17 (30%)

(n = 57).

*14 subjects used both opiate and cocaine based on self-report or urine toxicology at time of plasma sampling.

**Figure 1:**
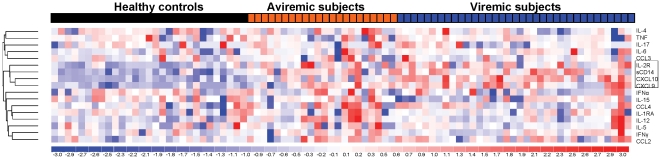
Plasma CXCL9, CXCL10, sIL-2R, and sCD14 represent a biomarker cluster that distinguishes HIV subjects from healthy uninfected controls. (A) Hierarchical clustering (supervised only by sample) by Euclidean distance and average linkage of 17 plasma biomarkers in 57 HIV patients and 29 healthy uninfected controls shows increased plasma levels and clustering of CXCL9, CXCL10, sIL-2R, and sCD14 (highlighted in box) in HIV subjects compared to healthy controls. In heat maps, red and blue represent increased and decreased levels relative to the mean level of a biomarker, respectively. Each column and row defines individual patient and biomarkers, respectively.

To identify an inflammatory biomarker profile that best distinguishes HIV patients from uninfected healthy controls, we analyzed expression levels of 17 biomarkers across all HIV-infected subjects and healthy controls by univariate analysis and semi-supervised hierarchical clustering. Levels of 7 inflammatory biomarkers (CXCL9, CXCL10, sIL-2R, CCL4, sCD14, IL-6, and CCL2) were higher in HIV+ subjects compared to healthy control subjects (FC≥1.3, p<0.05, FDR 0) (Table 2). Two chemokines (CXCL9, CXCL10), the T cell activation marker soluble IL-2 receptor (sIL-2R), and monocyte activation marker sCD14 were the top-ranked biomarkers based on p-values (FC 1.46 – 3.71, p<0.0001) that distinguished HIV subjects from controls. Additional biomarkers elevated in HIV subjects compared to controls were CCL4, IL-6, IL-12, and CCL2 (FC 1.19 - 1.61, p<0.05) (Table 2). Hierarchical clustering identified a biomarker cluster consisting of increased CXCL9, CXCL10, sIL-2R, and sCD14 that distinguished HIV-infected subjects from healthy controls (Figure 1). Two additional biomarker clusters consisted of CCL2 and IFNγ, and IL-12, IL-1RA, and CCL4, but neither distinguished HIV from control subjects (Figure 1). Thus, CXCL9, CXCL10, sIL-2R, and sCD14 represent a plasma biomarker cluster that distinguishes HIV subjects from healthy controls.

**Table 2:** Plasma biomarkers that distinguish all HIV subjects, viremic HIV subjects, and aviremic HIV subjects, from healthy controls.

Cytokines/Chemokines	HIV subjects versus controls (n = 57)		Viremic subjects versus controls (n = 35)		Aviremic subjects versus controls (n = 22)	
	FC	p value	FC	p value	FC	p value
**CXCL9**	**3.71**	**<0.0001**	**4.69**	**<0.0001**	**3.07**	**<0.0001**
**CXCL10**	**3.25**	**<0.0001**	**4.26**	**<0.0001**	**2.03**	**<0.0001**
**sIL-2R**	**2.22**	**<0.0001**	**2.38**	**<0.0001**	**2.25**	**<0.001**
CCL4	1.61	0.0267*	1.67	0.0188*	1.91	0.0441*
**sCD14**	**1.46**	**<0.0001**	**1.53**	**<0.0001**	**1.40**	**<0.0001**
IL-6	1.35	0.0143*	1.45	0.0081*	1.27	0.1861
CCL2	1.35	0.0475*	1.45	0.0179*	1.36	0.2110
IL-12	1.19	0.0071*	1.11	0.1380	1.36	0.0037*

*Shown are the 8 analytes that demonstrated significant differences in at least one inter-group comparison using the following criteria: mean fold change (FC)≥1.3, and p<0.05 by unpaired t-test;*

*difference is statistically significant (p<0.05); Comparisons with bold fonts are top ranked biomarkers (p<0.0001 and p<0.001).

### Plasma CXCL9, CXCL10, sIL-2R, and sCD14 levels distinguish both viremic and aviremic HIV subjects on cART from controls

**Figure 2:**
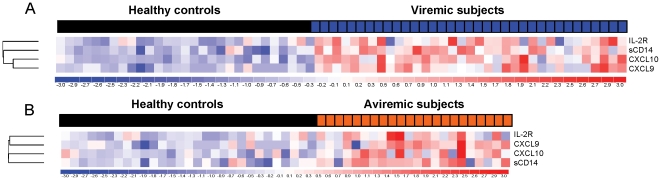
Increased plasma levels of CXCL9, CXCL10, sIL-2R, and sCD14 distinguish viremic and aviremic HIV subjects from controls. Supervised hierarchical clustering of plasma CXCL9, CXCL10, sIL-2R, and sCD14 levels measured in viremic (panel A) or aviremic, (panel B) HIV subjects compared to healthy controls. In heat maps, red and blue represent increased and decreased levels relative to the mean level of a biomarker, respectively. Each column and row defines individual patient and biomarkers, respectively.

To determine whether increased CXCL9, CXCL10, sIL-2R, and sCD14 levels can distinguish HIV patients on cART from uninfected controls regardless of the level of viral control in plasma, we performed univariate analysis and hierarchical clustering across subjects segregated according to either detectable (>400 HIV RNA copies/ml) or undetectable (<400 copies/ml) plasma VL. The viremic and aviremic groups had similar median age and nadir CD4 counts, but median current CD4 counts were lower in viremic compared to aviremic subjects (50 versus 152 cells/µl, p<0.001). The four biomarkers (CXCL9, CXCL10, sIL-2R, and sCD14) identified in the preceding analysis were significantly increased in both the viremic and aviremic groups (FC 1.5–4.7 and 1.4–3.1, respectively, p<0.001) compared to healthy controls, distinguishing both groups from healthy controls in supervised analyses (Table 2 and Figure 2). By univariate analysis, we also identified additional plasma biomarkers increased in viremic (CCL2, CCL4, and IL-6) or aviremic (IL-12 and CCL4) subjects compared to controls (FC≥1.3, p<0.05, FDR 0%) (Table 2) and found higher IL-12 levels in aviremic (FC 1.36, p = 0.003) but not viremic (FC 1.11, p = 0.138) subjects compared to controls. CXCL9, CXCL10, sIL-2R, and sCD14 levels were slightly higher in viremic than in aviremic subjects, and IL-12 levels were slightly higher in aviremic than in viremic subjects (Table 2). These differences in cytokine/chemokine levels between the viremic and aviremic groups were less than expected, probably reflecting the selection of subjects at late stages of disease. Unsupervised hierarchical clustering based on CXCL9, CXCL10, sIL-2R, and sCD14 levels across viremic, aviremic, and control subjects yielded low rate of misclassification corresponding to 20% (7/35) of viremic (Figure S1, panel A) and 18% (4/22) of aviremic subjects (Figure S1, panel B). The accuracy of unsupervised clustering for correctly predicting classification of HIV versus control subjects was not improved by adding any other biomarker, alone or in combination, to the analysis. Thus, CXCL9, CXCL10, sIL-2R, and sCD14 represent a combinatorial inflammatory biomarker panel that distinguishes both viremic and aviremic HIV patients on cART from healthy controls with ∼80% accuracy, and its predictive accuracy for classifying patients by unsupervised clustering is not improved by adding additional cytokine/chemokine biomarkers.

### Plasma CXCL9, CXCL10, sIL-2R, and sCD14 explain most of the variance between all HIV or aviremic HIV subjects and controls

To add another layer of dimensionality to analysis of the data set, we performed PCA (unsupervised analysis) and PLS-DA (supervised analysis), an approach that can explain the variance in the data by reducing the number of dimensions, thereby revealing internal structure of the data. First, we performed PCA, which finds the directions of maximum variance in a dataset without referring to class labels. PCA applied to 17 plasma biomarkers revealed that the first 3 components explain 50.7% of the variance between HIV subjects and controls along the axis defined by PC1, PC2, and PC3, with CXCL9, CXCL10, sIL-2R, and sCD14 clustering together in loading plots (data not shown). However, PCA did not accurately segregate all HIV or aviremic HIV subjects from controls.

**Figure 3:**
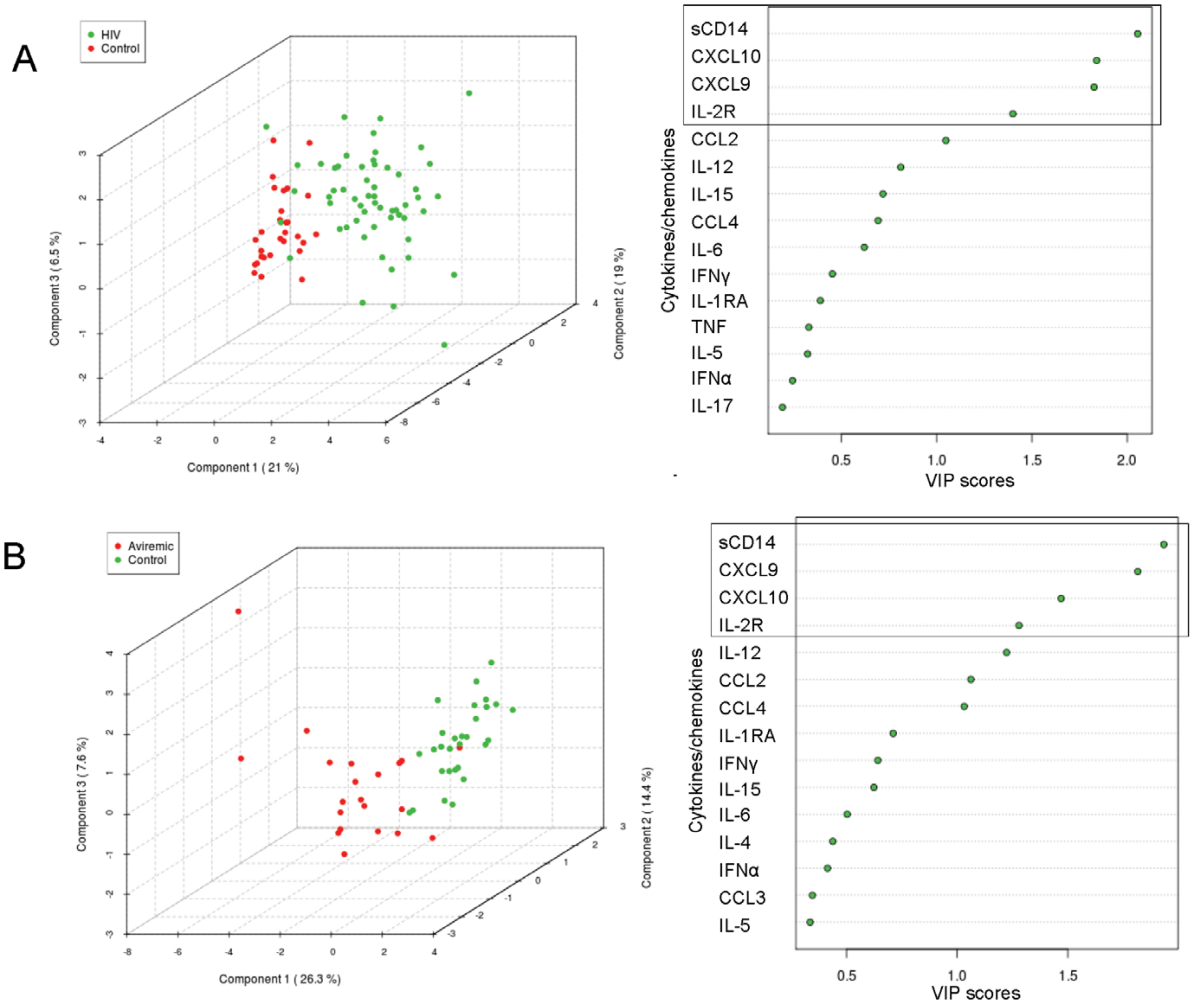
PLS-DA of 17 biomarkers shows separation of HIV subjects and aviremics from controls and identifies top-ranked biomarkers accounting for separation between groups. (A) PLS-DA represented as a three dimensional scatter plot shows the top 3 components in the matrix of biomarker data (n = 17 biomarkers) measured in HIV (green dots, n = 57) and uninfected control subjects (red dots, n = 29). 46.5% of the variance observed in the matrix of biomarker data is explained by the first 3 components. Plot on the right shows variables important in projection (VIP) plot ranking sCD14, CXCL9, CXCL10, and sIL-2R as the top 4 biomarkers accounting for the variance between all HIV subjects and controls. (B) PLS-DA represented as a three dimensional scatter plot showing the top 3 components in the matrix of biomarker levels measured in aviremic HIV subjects (red dots, n = 22) and uninfected control samples (green dots, n = 29). 48.3% of the variance in the matrix of biomarkers is explained by the first 3 components. Plot on the right represents VIP plot ranking sCD14, CXCL9, CXCL10, and sIL-2R as the top 4 biomarkers explaining the variance between aviremic HIV subjects and uninfected controls.

Next, we analyzed the data using PLS-DA, a supervised classification method based on the partial least squares (PLS) approach that uses multiple linear regression analysis to find the direction of maximum covariance between a dataset and class membership and prioritize features that contribute significantly to class prediction. A permutation test (2000 permutations) was then applied to determine the importance of class separation in the dataset. When PLS-DA was applied to 17 plasma biomarkers and data from all 57 HIV subjects were projected onto these 3 components, HIV and control subjects could be separated along the axis defined by 3 components explaining 46.5% of the variance (Figure 3A). Permutation tests showed significant separation distance between the 2 groups (p<0.0005). Similar analysis applied to the aviremic group showed that aviremic HIV subjects and controls could be separated along the axis defined by 3 components explaining 48.3% of the variance in the matrix of 17 plasma biomarkers (Figure 3B). Permutation tests confirmed significant separation between the 2 groups (p<0.0005). Features that contribute significantly to class prediction were then ranked using variables important in projection (VIP) scores, which are based on the weighted coefficients of the PLS model; VIP scores above a threshold of 1 are considered important. VIP score plots revealed sCD14, CXCL10, CXCL9, and sIL-2R as the top 4 biomarkers explaining the class separation between all HIV or aviremic HIV subjects and controls (Figure 3). These results provide additional evidence that CXCL9, CXCL10, sIL-2R, and sCD14 represent a combinatorial plasma inflammatory biomarker signature that distinguishes both viremic and aviremic HIV patients on cART from uninfected controls.

### Relationship of biomarkers in the combinatorial signature to interferons and HIV disease markers

**Figure 4:**
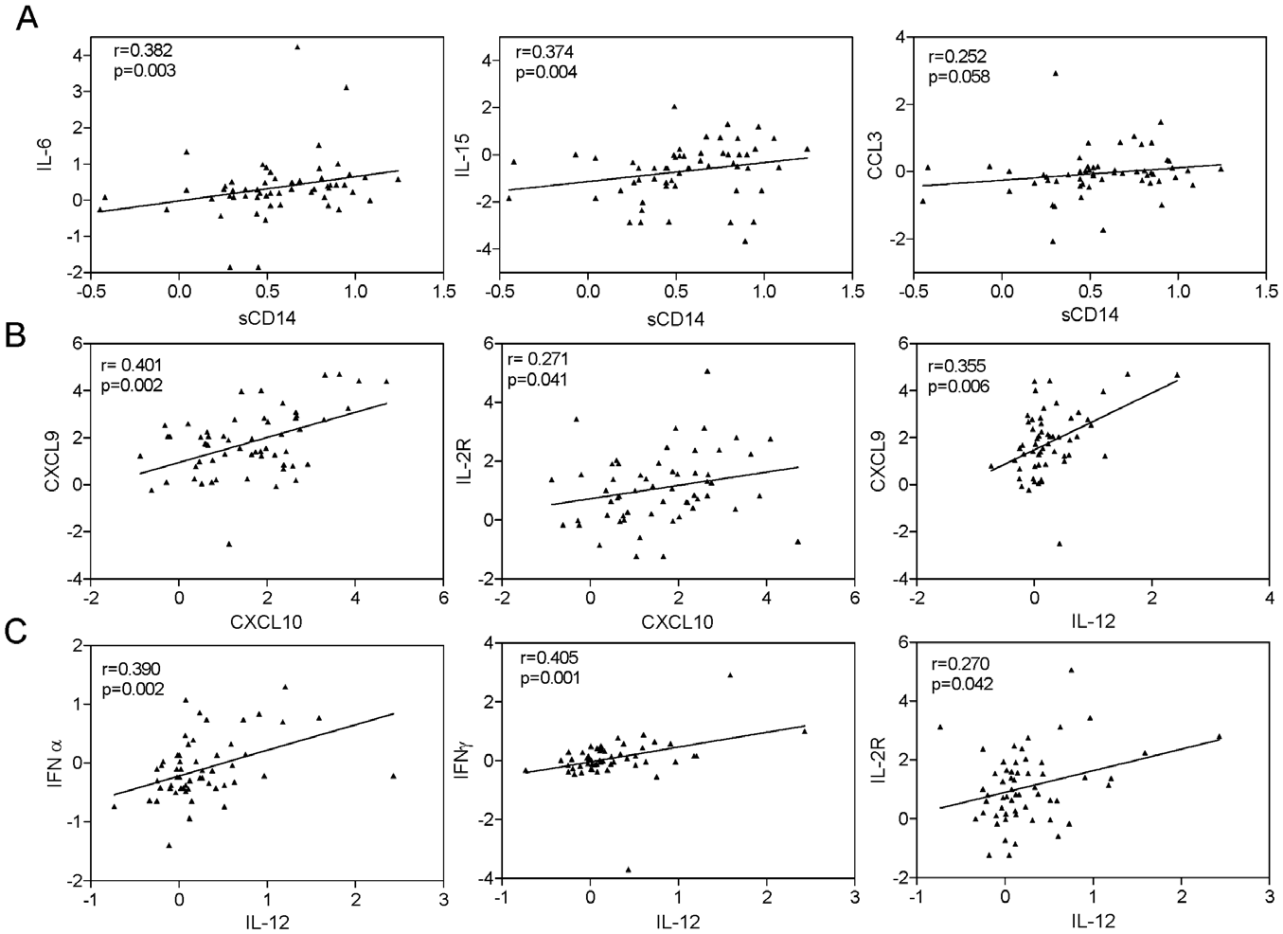
Inter-relationships between plasma inflammatory biomarkers and interferons in HIV subjects on cART. (A) Plasma sCD14 shows positive correlation with IL-6, IL-15, and trend towards significant correlation with CCL3. (B) CXCL10 correlated positively with CXCL9 andsIL-2R, while IL-12 was significantly associated with CXCL9. (C) IL-12 correlated positively with IFNα, IFNγ, and sIL-2R. Shown are log2 transformed values of measurements normalized to the mean of healthy controls. Data was analyzed by Spearman correlation, with p<0.05 considered significant.

To examine relationships between individual components of the four-marker signature identified in the studies described above and interferons or other biomarkers in HIV subjects (n = 57), we performed Spearman correlation analysis. We found the following correlations: 1) plasma sCD14 correlated with IL-6 (p = 0.003) and IL-15 (p = 0.004), and trended towards significance with CCL3 (p = 0.058) (Figure 4A); 2) CXCL9 correlated with CXCL10 and IL-12 (p = 0.002 and p = 0.006, respectively) (Figure 4B); 3) sIL-2R correlated with CXCL10 and IL-12 (p = 0.041 and p = 0.042 respectively, Figure 4B and C). We examined the relationship of type I/II IFN levels to other biomarkers and found that IFNα and γ correlated with IL-12 (p = 0.002 and p = 0.001, respectively; Figure 4C). IFNα also correlated with CCL4 and CCL2 (r = 0.400, p = 0.002 and r = 0.291, p = 0.027, respectively, data not shown). These findings imply a close relationship between type I and II IFNs, IL-12, and individual components of the inflammatory biomarker signature.

**Figure 5:**
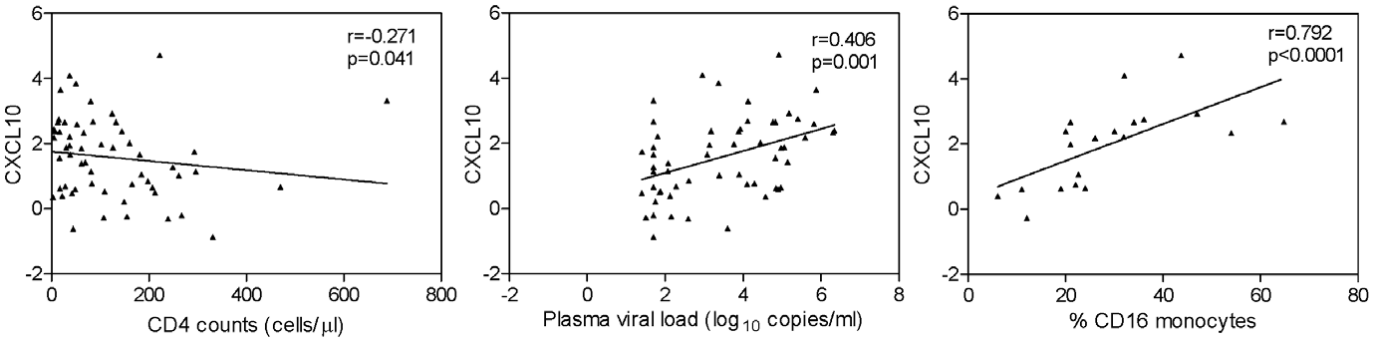
Relationships between plasma CXCL10 levels and CD4 count, plasma viral load, and frequency of CD16+ monocytes. Plasma CXCL10 levels show positive correlation with plasma viral load and frequency of CD16+ monocytes, and negative correlation with CD4 counts. Data was analyzed by Spearman correlation, with p<0.05 considered significant.

We then examined the relationship between individual biomarkers within the combinatorial biomarker signature and HIV disease markers (CD4 count, plasma VL, and LPS). This analysis revealed that only CXCL10 showed significant correlations with disease markers, in particular an inverse correlation with CD4 count and positive correlation with plasma VL (r = −0.271, p = 0.041 and r = 0.406, p = 0.001, respectively) (Fig. 5). Because previous studies demonstrated an association between expansion of the proinflammatory CD16+ monocyte subset and HIV disease progression [29], [33], [34], [35], [36], we also examined the relationship between signature biomarkers and frequency of the CD16+ monocyte subset using data for 20 HIV-infected subjects available from our previous study [29]. We found a positive correlation between CXCL10 levels and frequency (%) of CD16+ monocytes (p<0.0001, Figure 5, right panel). These findings identify CXCL10 as a component of the plasma inflammatory biomarker signature we identified in HIV subjects that is strongly associated with disease markers (high plasma VL and low CD4 count) and increased frequency of CD16+ monocytes.

To further examine inter-relationships between biomarkers as well as relationships between biomarkers and HIV disease markers (CD4 count and plasma VL), we performed similar analyses for viremic and aviremic HIV subjects. In viremic subjects, sCD14 correlated with IFNγ and IL-15 (r = 0.41, p = 0.014 and r = 0.403, p = 0.016, respectively), while CXCL9 correlated with IL-12 and CXCL10 (r = 0.357, p = 0.034; r = 0.468, p = 0.004, respectively) and IL-12 correlated with IFNα and IFNγ (r = 0.435, p = 0.008 and r = 0.544, p = 0.0007, respectively). In aviremic subjects, sCD14 levels correlated with IL-6 (r = 0.530, p = 0.012), while CXCL9 correlated with IL-12 and CCL2 (r = 0.528, p = 0.011 and r = 0.572, p = 0.005, respectively) and IFNα correlated with CCL4 and CCL2 (r = 0.540, p = 0.009 and r = 0.648, p = 0.001, respectively). However, none of the individual biomarker components of the four-marker signature was associated with HIV disease markers in either group. In particular, correlations between CXCL10 and disease markers found in the studies describe above for the total cohort of 57 HIV subjects (Fig. 5) were lost when similar analyses were performed for the viremic and aviremic subgroups. This difference may reflect insufficient statistical power for this analysis given the smaller sample sizes of these subgroups (n = 35 and 22, respectively). These findings demonstrate a close relationship between type I and II interferons, IL-12, and monocyte-derived inflammatory cytokines/chemokines in both viremic and aviremic HIV subjects on cART.

### Cocaine abuse in HIV-infected subjects is associated with biomarkers indicative of increased T cell activation

To explore relationships between plasma biomarker levels and clinical subgroups, we analyzed biomarker levels in subjects grouped according to presence or absence of a HIV-associated neurocognitive disorders (HAND) clinical diagnosis, HCV co-infection, and active use of cocaine or heroin. Unsupervised hierarchical clustering identified a large cluster of subjects with active cocaine abuse (primarily via intravenous route), representing 12/17 cocaine users with higher levels of sIL-2R, sCD14, CXCL9, CXCL10, CCL4, and CCL2 compared to non-users (p = 0.0005; data not shown), while no significant clustering was associated with HAND or HCV co-infection. Therefore, we next examined whether any unique plasma biomarker expression pattern is associated with cocaine abuse defined by self report on PRISM or CIDI interviews together with positive urine toxicology. Comparing biomarker levels between these two groups using dChip software revealed that sIL-2R was increased, while IL-17 was decreased (FC 2.6 and p = 0.012 and FC −1.5 and p = 0.046, respectively; FDR 0.0) in viremic subjects with active cocaine use and positive urine toxicology at time of plasma sampling versus non-users with no self-reported cocaine/opiate usage and negative urine toxicology (n = 16; 8 subjects per group) (data not shown). Furthermore, PLS-DA identified sIL-2R and IL-17 as top-ranked biomarkers accounting for the separation between these two groups based on VIP scores (Figure S2). These two groups had similar median age, current and nadir CD4 counts, and mean plasma VL. Together, these findings suggest that active cocaine abuse is associated with markers indicative of T cell activation (higher sIL-2R), and possibly depletion of the Th17 cells (lower IL-17), in HIV-infected patients on nonsuppressive cART.

## Discussion

By multiplex profiling, we identified a combinatorial inflammatory biomarker signature in plasma consisting of 2 interferon-stimulated gene products (CXCL9, CXCL10), T cell activation marker (sIL-2R), and monocyte activation marker (sCD14) that distinguishes viremic and aviremic HIV patients on cART from uninfected controls. Increased CXCL10 correlated with low CD4 count and high plasma VL, consistent with previous studies in other HIV cohorts [37], [38]. We also found a positive correlation between CXCL10 and frequency of the proinflammatory CD16+ monocyte subset, a subset that expands during HIV disease progression. CXCL9, CXCL10, sIL-2R, and sCD14 were top-ranked biomarkers explaining most of the variance between all HIV subjects, or aviremic HIV subjects, and uninfected controls. Thus, CXCL9, CXCL10, sIL-2R, and sCD14 represents a plasma inflammatory biomarker signature that may be useful as a surrogate marker to monitor interferon responses and chronic immune activation in both viremic and aviremic HIV patients on cART.

Our identification of plasma CXCL9 and CXCL10 as plasma biomarkers associated with HIV infection is consistent with previous studies [7], [37], [39], [40], [41], and increased CXCL10 and IFNα were previously detected in HIV patients on cART [7], [19], [37], [41], [42]. Elevated CXCL10 is also detected in other chronic viral infections, including HCV [43], [44], [45], and HCV/HIV coinfection [46], [47], [48], suggesting a role in the immune response to viral infections. In the present study, we found significant associations between plasma CXCL9 and CXCL10, in addition to correlations of these chemokines with other inflammatory biomarkers including sIL-2R, IL-12, and CCL2. Consistent with previous studies [37], [38], CXCL10 correlated positively with plasma VL, and inversely with CD4 T cell count, suggesting a close relationship between CXCL10, HIV replication, and markers of disease progression. A novel finding was the correlation between CXCL10 and the frequency of CD16+ monocytes in HIV patients on cART. A recent study found that the percentage of CD14+CD16+ monocytes was significantly increased in PBMC from melanoma patients exposed to IFNα ex vivo. This study also found that increased CXCL10 induced by treatment with recombinant IFNα was paralleled by expansion of CD14+/CD16+ monocytes in vivo [49]. Another study reported increased plasma CXCL10 in melanoma patients treated with relatively low doses of IFNα associated with a trend towards increased frequency of CD16+ monocytes in vivo [50]. Together, these findings suggest that plasma CXCL10 is a biomarker closely associated not only with HIV disease markers but also with an increased frequency of CD16+ monocytes in vivo. The mechanism linking expansion of CD16+ monocytes to increased CXCL10 levels remains unclear and merits further investigation.

Because only plasma samples were available for the study, it was not possible to address the cellular sources of elevated plasma cytokines/chemokines. However, the biomarker pattern is indicative of activated monocytes, plasmacytoid dendritic cells (pDC), and myeloid dendritic cells (mDC), being likely sources. pDC are the primary source of IFNα in HIV-infected individuals, producing up to 1000-fold more than other cell types [10], [51], which in turn drives monocyte activation and monocyte production of interferon-induced cytokines/chemokines including CXCL9, CXCL10, and CCL2 [19]. CXCL9 and CXCL10, produced mainly by activated monocytes/macrophages and dendritic cells (both pDC and mDC), recruit CXCR3+ effector and memory CD8 T cells and NK cells to sites of inflammation [8], [10], [40], [52]. IL-12 and CCL4 were elevated in viremic and aviremic patients compared to controls, and IL-12 correlated with IFNα, IFNγ, CXCL9, and sIL-2R. These findings, together with identification of the plasma biomarker signature consisting of CXCL9, CXCL10, sIL-2R, and sCD14 and associations between these biomarkers and elevated IL-12, produced mainly by mDC, imply close relationships between persistent elevation of type I and II interferons, IL-12, monocyte- and dendritic cell-derived inflammatory cytokines/chemokines, and chronic activation of T cells and monocytes in HIV patients on cART.

In the present study, plasma IL-12 was higher in aviremic than in viremic HIV subjects. This finding is consistent with a recent study [53] that showed an association between IFNα production by pDC and IL-12 production by mDC in HIV patients with CD4 counts >200 cells/µl during cART therapy and higher DC production of IL-12 at weeks 6 and 12 on cART in patients with CD4 counts >200 cells/µl compared to those with CD4 counts <200/µl. Consistent with previous studies [53], [54], we found an association between IL-12 and IFNα and -γ in the study cohort of HIV patients, and with CCL2, CCL4 in aviremic patients on cART. In contrast, a recent study reported that untreated progressive HIV infection was associated with increased CXCL10 and TNF, but decreased IL-12 and IL-15 [37], which may reflect differences in the clinical characteristics of study subjects. Unexpectedly, we found no significant difference in IFNα and IFNγ levels between HIV patients and controls, which may reflect the wide range of IFN levels among healthy controls. Nonetheless, the associations of IFNα, IFNγ, CXCL9, and sIL-2R with IL-12, together with previous studies demonstrating that IL-12 augments IFNγ responses, suggest that IL-12-mediated stimulation of IFNγ responses may be a factor contributing to T cell activation in HIV patients on cART.

Consistent with prior studies, plasma sCD14 correlated not only with IL-6 [18], [55], but also with IL-15 [22], [56]. Elevated IL-15 is associated with higher plasma VL during acute HIV infection [38], while IL-15 is expressed at higher levels in monocytes from HIV-infected long-term nonprogressors compared to progressors or uninfected controls [57]. IL-15 is a Th1 cytokine expressed on monocytes upon activation that shares activities with IL-2, such as stimulation of T cell proliferation and activation, but also stimulates cell adhesion and production of proinflammatory cytokines [58] and NK cell functions, including secretion of IFNγ and CCR5 ligands [59], [60]. Excess IL-15 expression on monocytes leads to increased MHC II expression, contributing to activation or proliferation of T cells in diseases such as rheumatoid arthritis [61]. In vitro priming of NK cells with IL-15 enhances secretion of IFNγ and CC chemokines in viremic and aviremic HIV patients [54]. Thus, IL-15 has complex immunomodulatory effects that can be beneficial, as well as pathogenic [61], [62], [63], [64], [65].

Crack cocaine use is associated with accelerated progression to AIDS [66], [67], but the mechanisms underlying this association are unclear. Previous studies suggest that cocaine abuse may affect HIV pathogenesis by causing an imbalance of Th1-Th2 cytokines and stimulation of IFNγ responses [68], [69] and by upregulating HIV coreceptors [70], possibly by acting through σ-1 receptors [70], [71]. Consistent with this model, cocaine-mediated upregulation of IFNγ has been demonstrated in cocaine-dependent subjects following intravenous cocaine infusion [69]. Our finding that a cluster of viremic cocaine users had elevated sIL-2R, CXCL9, CXCL10, CCL2, and CCL4 compared to other viremic subjects is consistent with augmentation of interferon responses. Furthermore, PLS-DA and VIP scores identified upregulated sIL-2R and downregulated IL-17 as top-ranked biomarkers accounting for separation between viremic HIV subjects with versus without active cocaine abuse. Downregulation of IL-17 could relate to cocaine use via IFNγ-mediated induction of indoleamine 2, 3-dioxygenase (IDO) and tryptophan depletion in activated monocytes/macrophages, a pathway previously linked to increased immune activation in HIV-infected individuals abusing cocaine and other stimulants [72], [73]. IDO regulates the Th17/Treg cell balance, with induction of IDO activity resulting in a relative increase in Tregs and decrease in Th17 cells [74], [75]. These findings raise the possibility that cocaine abuse may augment T cell activation and promote Th17 cell depletion in treated HIV patients via direct or indirect effects of cocaine on IFNγ-mediated pathways involving IDO and tryptophan metabolism.

We acknowledge several limitations to this study, including the cross-sectional study design and small sample size, which may have decreased the power to detect significant associations between biomarker expression and disease markers or clinical subgroups. Also, the narrow selection criteria used to define the study cohort (CD4 nadir <300) limit our findings to those with advanced HIV disease and may also explain why biomarker levels were only slightly higher in viremic than in aviremic HIV subjects. The study cohort was from NNTC, which specifically recruits individuals with advanced disease, and Lemuel Shattuck Hospital, which treats a large population of HIV patients with advanced disease, to represent a diverse population of HIV-infected individuals with broad range of viral loads. Therefore, results cannot be generalized to all populations. Indeed, the same cohort was used to derive the plasma biomarker signature and examine its classification accuracy for predicting HIV versus control subjects (∼80% accuracy). As such, we do not know if the classification accuracy may be overestimated until the signature is examined in additional cohorts. The multiplex bead assay provides a wealth of expression data, but we are unable to conclude which cell types are responsible for production of individual biomarkers. In view of these limitations, future studies should utilize flow cytometric assays along with multiplex assays and longitudinal cohorts including subjects at earlier stages of disease to better define relationships between the plasma biomarker signature identified in the present study, chronic immune activation, and clinical endpoints.

### Conclusions

We identified a plasma inflammatory biomarker signature consisting of 2 interferon stimulated gene products (CXCL9, CXCL10), T cell activation marker (sIL-2R), and monocyte activation marker (sCD14) that may be clinically useful as a surrogate marker of immune activation in both viremic and aviremic HIV patients on cART. Increased levels of the interferon-induced chemokines CXCL9 and CXCL10 implicate activation of IFN responses, while sIL-2R and sCD14 are markers of T cell and monocyte activation, respectively. CXCL10 correlates with several disease markers including high plasma VL, low CD4 counts, and expansion of the CD16+ monocyte subset. Our analysis of IL-12 and other cytokine/chemokine biomarkers implies a close relationship between persistent elevation of type I and II interferons, IL-12, monocyte- and dendritic cell-derived inflammatory cytokines/chemokines, and activation of T cells and monocytes in HIV patients on cART. Further studies are warranted to understand the clinical utility of this combinatorial plasma inflammatory biomarker signature as a surrogate marker to monitor immune activation and therapeutic responses in HIV patients on cART with diverse clinical phenotypes.

## Supporting Information

Table S1 **Plasma biomarker levels for all HIV subjects and healthy controls, and clinical characteristics for HIV subjects.**
(XLS)

Figure S1 **Unsupervised hierarchical clustering of plasma CXCL9, CXCL10, sIL-2R, and sCD14 levels segregates viremic and aviremic HIV subjects from controls with ∼80% accuracy.** (A) Unsupervised hierarchical clustering was performed by average linkage and Euclidean distance on 4 biomarkers (CXCL9, CXCL10, and sIL-2R and sCD14) across viremic (A, blue boxes) and aviremic (B, orange boxes) HIV subjects and healthy controls (black boxes). Analysis was run across the covariates defining clinical group (A, viremic versus controls; B, aviremic versus controls), plasma VL, and current and nadir CD4 count. In heatmaps, red represents increased levels and blue represents decreased levels relative to the mean levels of a biomarker. Each column and row defines individual patients and biomarkers, respectively. The analysis shows a low rate of misclassification (7/35, corresponding to 20%, and 4/22, corresponding to 18% for viremic and aviremic HIV subjects, respectively) based on unsupervised hierarchical clustering.
(TIF)

Figure S2 **Inflammatory biomarkers separate viremic HIV subjects testing positive for cocaine from non-users in PLS-DA.** PLS-DA represented as three dimensional scatter plot (left panel) shows the top 3 components of biomarker levels measured in viremic HIV subjects with active cocaine use (with positive urine toxicology) (red triangles, n = 8) and non-users (green triangles, n = 8). Plot shows that 57.8% of the variance in the matrix of biomarkers is explained by the first 3 components. Variables important in projection (VIP) plot (right panel) ranks sIL-2R and IL-17 as the top 2 biomarkers accounting for separation between viremic HIV subjects with active cocaine use and non-users.
(TIF)
