# Supplementary figures and images for: A Plasma Biomarker Signature of Immune Activation in HIV Patients on Antiretroviral Therapy
Source: PLoS One. 2012 Feb 17;7(2):e30881. doi: 10.1371/journal.pone.0030881 (PMC3281899; doi:10.1371/journal.pone.0030881)

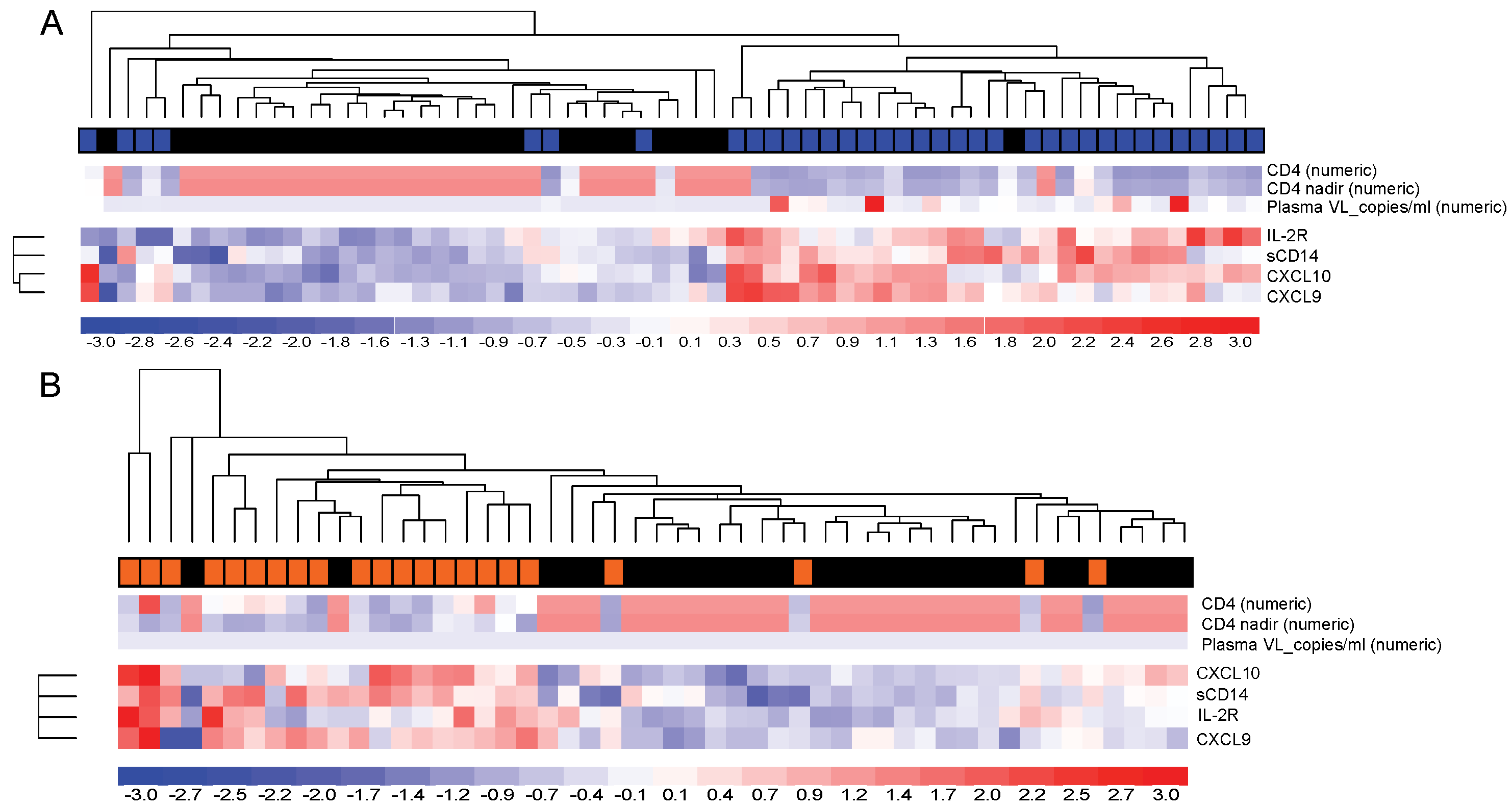

Supplement: Figure S1 — Unsupervised hierarchical clustering of plasma CXCL9, CXCL10, sIL-2R, and sCD14 levels segregates viremic and aviremic HIV subjects from controls with ∼80% accuracy. (A) Unsupervised hierarchical clustering was performed by average linkage and Euclidean distance on 4 biomarkers (CXCL9, CXCL10, and sIL-2R and sCD14) across viremic (A, blue boxes) and aviremic (B, orange boxes) HIV subjects and healthy controls (black boxes). Analysis was run across the covariates defining clinical group (A, viremic versus controls; B, aviremic versus controls), plasma VL, and current and nadir CD4 count. In heatmaps, red represents increased levels and blue represents decreased levels relative to the mean levels of a biomarker. Each column and row defines individual patients and biomarkers, respectively. The analysis shows a low rate of misclassification (7/35, corresponding to 20%, and 4/22, corresponding to 18% for viremic and aviremic HIV subjects, respectively) based on unsupervised hierarchical clustering. (TIF) [file pone.0030881.s002.tif]

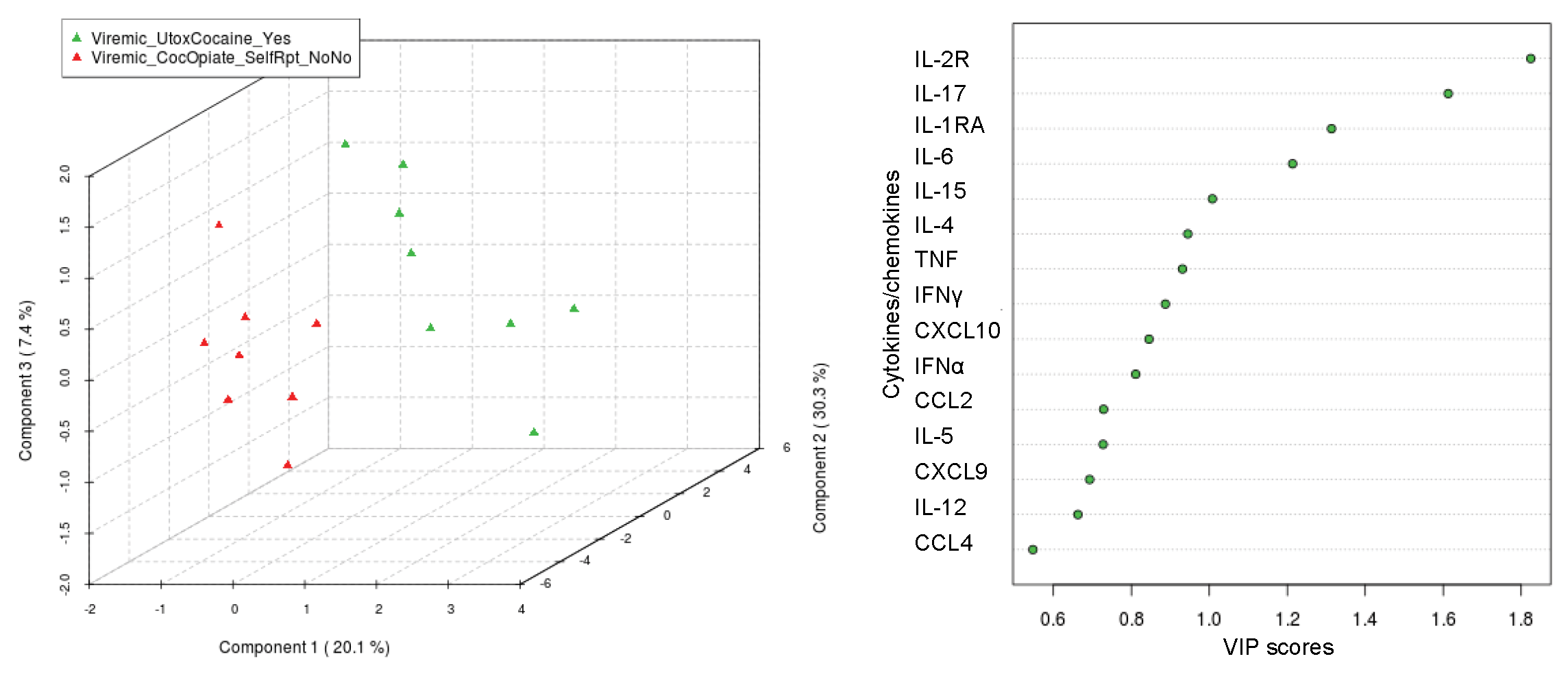

Supplement: Figure S2 — Inflammatory biomarkers separate viremic HIV subjects testing positive for cocaine from non-users in PLS-DA. PLS-DA represented as three dimensional scatter plot (left panel) shows the top 3 components of biomarker levels measured in viremic HIV subjects with active cocaine use (with positive urine toxicology) (red triangles, n = 8) and non-users (green triangles, n = 8). Plot shows that 57.8% of the variance in the matrix of biomarkers is explained by the first 3 components. Variables important in projection (VIP) plot (right panel) ranks sIL-2R and IL-17 as the top 2 biomarkers accounting for separation between viremic HIV subjects with active cocaine use and non-users. (TIF) [file pone.0030881.s003.tif]
